# Antibiotic Resistance Profiles and Molecular Characteristics of Extended-Spectrum Beta-Lactamase (ESBL)-Producing *Escherichia coli* and *Klebsiella pneumoniae* Isolated From Shrimp Aquaculture Farms in Kerala, India

**DOI:** 10.3389/fmicb.2021.622891

**Published:** 2021-08-19

**Authors:** Gopalan Krishnan Sivaraman, Vineeth Rajan, Ardhra Vijayan, Ravikrishnan Elangovan, Alison Prendiville, Till T. Bachmann

**Affiliations:** ^1^Microbiology, Fermentation and Biotechnology Division, ICAR-Central Institute of Fisheries Technology, Kochi, India; ^2^Department of Biochemical Engineering and Biotechnology, Indian Institute of Technology Delhi, New Delhi, India; ^3^London College of Communication, University of the Arts London, London, United Kingdom; ^4^Division of Infection and Pathway Medicine, Edinburgh Medical School, The University of Edinburgh, Edinburgh, United Kingdom

**Keywords:** shrimp aquaculture, extended-spectrum beta-lactamase, *Escherichia coli*, *Klebsiella pneumoniae*, multidrug resistance

## Abstract

This study was undertaken to evaluate the prevalence of extended-spectrum beta-lactamase (ESBL)-producing *Escherichia coli* and *Klebsiella pneumoniae* in selected shrimp aquaculture farms (*n* = 37) in Kerala, South India and to characterize the isolates using molecular tools. Overall, a low prevalence of ESBL-producers was found in the farms, most likely due to the reduced antibiotic usage in the shrimp farming sector. Out of the 261 samples (77 shrimp and 92 each of water and sediment), 14 (5.4%) tested positive for ESBL-*E. coli* or ESBL-*K. pneumoniae*. A total of 32 ESBL-*E. coli* and 15 ESBL- *K. pneumoniae* were recovered from these samples. All ESBL isolates were cefotaxime-resistant with minimal inhibitory concentration (MIC) ≥32 μg/ml. Of all isolates, 9 (28.1%) *E. coli* and 13 (86.7%) *K. pneumoniae* showed simultaneous resistance to tetracycline, ciprofloxacin, and trimethoprim-sulfamethoxazole. PCR analysis identified CTX-M group 1 (*bla*_*CTX–M–15*_) as the predominant ESBL genotype in both *E. coli* (23, 71.9%) and *K. pneumoniae* (15, 100%). Other beta-lactamase genes detected were as follows: *bla*_*TEM*_ and *bla*_*SHV*_ (11 *K. pneumoniae*), *bla*_*CTX–M group 9*_ (9 *E. coli*), and *bla*_*CMY–2*_ (2 *E. coli*). Further screening for AMR genes identified *tetA* and *tetB* (13, 40.6%), *sul1* (11, 34.4%), *sul2* (9, 28.1%), *catA* and *cmlA* (11, 34.4%), *qepA* and *aac(6*′*)-Ib-cr* (9, 28.1%) and *strAB* and *aadA1* (2, 6.3%) in *E. coli*, and *qnrB* (13, 86.7%), *qnrS* (3, 20%), *oqxB* (13, 86.7%), *tetA* (13, 86.7%), and *sul2* (13, 86.7%) in *K. pneumoniae* isolates. Phylogenetic groups identified among *E. coli* isolates included B1 (4, 12.5%), B2 (6, 18.8%), C (10, 31.3%), D (3, 9.4%), and E (9, 28.1%). PCR-based replicon typing (PBRT) showed the predominance of IncFIA and IncFIB plasmids in *E. coli*; however, in *K. pneumoniae*, the major replicon type detected was IncHI1. Invariably, all isolates of *K. pneumoniae* harbored virulence-associated genes viz., *iutA*, *entB*, and *mrkD*. Epidemiological typing by pulsed-field gel electrophoresis (PFGE) revealed that *E. coli* isolates recovered from different farms were genetically unrelated, whereas isolates of *K. pneumoniae* showed considerable genetic relatedness. In conclusion, our findings provide evidence that shrimp aquaculture environments can act as reservoirs of multi-drug resistant *E. coli* and *K. pneumoniae.*

## Introduction

Antimicrobial resistance (AMR) is undoubtedly a huge public health crisis across the globe. AMR had long been regarded as an issue of human health alone, but recent years have witnessed a growing recognition of the imprudent use of antibiotics in multiple sectors (agriculture, food animals, aquaculture, and environment) as important drivers of resistance. However, our understanding of the extent and magnitude of the contribution of each sector to the overall burden of AMR is quite limited. Of particular concern are the intensive animal production practices, both in livestock and aquaculture sectors where antibiotics are regularly used for therapeutic, prophylactic or growth promotion purposes. Aquaculture is a rapidly growing food sector with the majority of production taking place in low and middle income countries (LMICs) (FAO, 2016). Global antimicrobial consumption in aquaculture in 2017 was estimated at 10,259 tons, with India (11.3%) being the second-largest consumer after China (Schar et al., 2020). In particular, shrimp aquaculture industry has gained prominence in recent decades owing to the growing demand in the global market. India was the top exporter of farmed shrimps in 2018 with the United States, Vietnam, and EU countries as the leading markets for Indian shrimps (FAO, 2019). This sector faces many challenges, the most important being the high disease burden caused by various viruses, bacteria, fungi, and parasites. Despite the fact that most of the devastating diseases of shrimps are due to viral or parasitic infections, a wide range of antibiotics are used in shrimp hatcheries and farms. There were recent incidences of rejection of Indian consignments of shrimp by EU countries owing to the presence of banned antibiotics such as furazolidone and chloramphenicol (Salunke et al., 2020). Antibiotics, when used appropriately, i.e., on the basis of proper diagnosis, can be helpful. But this does not seem to happen in most of the farms as farmers usually have poor access to diagnostic facilities. Moreover, the decision to use antibiotics in farms is often influenced by the advice given by neighboring farmers, drug suppliers, feed companies, private veterinarians and others.

Many factors are known to favor the emergence of AMR in aquaculture and its spread to other sectors. This includes high stocking densities leading to elevated stress and infections in shrimp, widespread use of various chemicals, nutrient-rich environment in the ponds, occupational human exposure to AMR bacteria, release of untreated water/waste to local environment, etc. (Thornber et al., 2020). Antibiotic-resistant bacteria, including human and zoonotic pathogens have been reported from various aquaculture settings (Cabello et al., 2013; Miranda et al., 2013; Watts et al., 2017). Among these, members of *Enterobacteriaceae* are of particular concern owing to their considerable ability to acquire resistance to various antimicrobials and to disseminate widely. Multidrug resistance in *Enterobacteriaceae* has become an escalating problem in healthcare as well as community settings worldwide. This in large part is due to the highly diverse and rapidly evolving group of beta-lactamases such as extended-spectrum beta-lactamases (ESBLs) and carbapenemases. ESBLs, generally found in *Enterobacteriaceae* and *Pseudomonas aeruginosa*, are a class of enzymes conferring resistance to penicillins, first-, second- and third-generation cephalosporins, and aztreonam, and are usually inhibited by beta-lactamase inhibitors such as clavulanic acid (Paterson and Bonomo, 2005). Enzyme families with ESBL phenotype are mainly described in class A (TEM, SHV, CTX-M, GES, and VEB families) and class D (OXA family) beta-lactamases (Paterson and Bonomo, 2005). Most of the ESBLs prevalent initially were TEM or SHV variants possessing amino acid substitutions which changed their substrate profile to include extended-spectrum cephalosporins. Currently, there are 183 variants of TEM and 178 variants of SHV enzymes, although not all of them are ESBLs (Bush and Bradford, 2020). In contrast, the CTX-M type ESBLs originated by the mobilization of chromosomal *bla* genes of *Kluyvera* spp., an innocuous rhizosphere bacterium (D’Andrea et al., 2013). Since 2000, CTX-M type enzymes gained prominence over other ESBLs and disseminated widely around the world resulting in a “CTX-M pandemic” with *Escherichia coli* being the predominant pathogen producing these enzymes (Cantón et al., 2012). AmpC beta-lactamases, another group of enzymes classified as class C beta-lactamases, possess broader hydrolytic spectrum (including cephamycins), and are not inhibited by class A enzyme inhibitors such as clavulanate.

Apart from their widespread occurrence in clinical settings, ESBL/AmpC-producing bacteria are increasingly being reported from livestock, companion animals, and environmental sources (Ewers et al., 2012; Haberecht et al., 2019; Hordijk et al., 2019). Wide dissemination of AMR genes in *Enterobacteriaceae* is mainly achieved by plasmids belonging to various incompatibility groups (Inc) such as F, A/C, L/M, I1, HI2, and N (Zurfluh et al., 2015). Though human to human transmission is shown to be the major route of transfer of these genes, the complex dynamics involved in the dissemination of AMR genes underpins the importance of continuously monitoring non-human sources for potential events of transmission to humans (Mughini-Gras et al., 2019).

With respect to aquaculture settings, very little data is available, particularly from this geographical region, on the prevalence of ESBL-producing bacteria. Thus, we undertook the present study with an aim to screen shrimp, water, and sediment samples from different shrimp farms in Kerala for ESBL-producing *Escherichia coli* and *Klebsiella pneumoniae*, and to further characterize them using molecular methods.

## Materials and Methods

### Study Sites and Sample Collection

**FIGURE 1:**
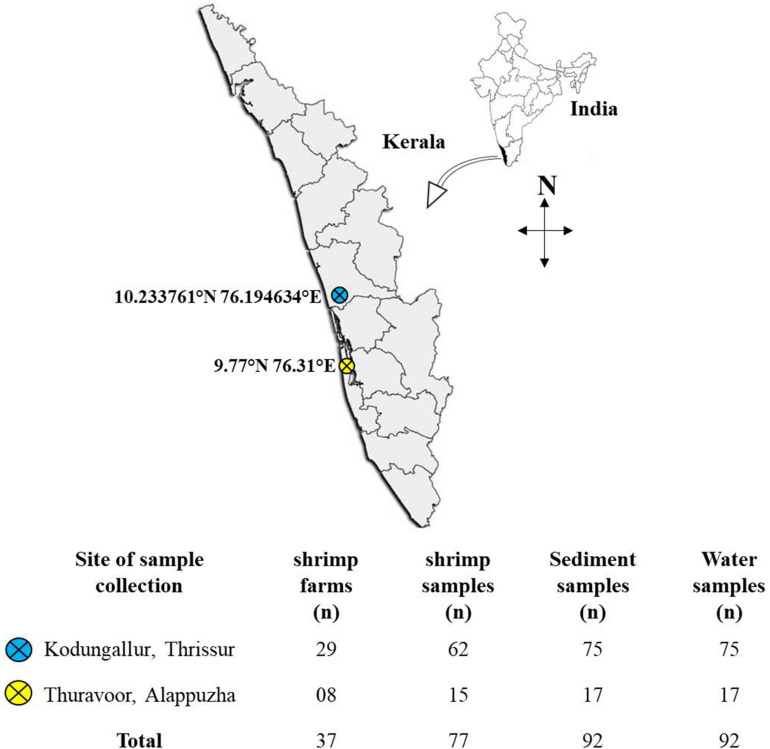
Map of the study region showing the locations of sampling sites. Below the map is a table giving information on the number of farms surveyed and the samples collected.

Sampling was done during the period November 2018–January 2020 at randomly selected shrimp aquaculture farms in two major shrimp farming zones in the state of Kerala: Kodungallur (in Thrissur district) and Thuravoor (in Alappuzha district) (Figure 1). For each farm, site description data viz., GPS co-ordinates, size of the farm, number of ponds, type of the cultured species, etc., were collected. Also, interviews were carried out with farmers as part of design ethnography to better understand the daily practices followed in the farms and also to identify the challenges for farmers within the system. This included information on seed procurement, feed types and feeding routine, antibiotic usage, natural remedies used in disease management, recording and testing practices, etc.

A total of 37 farms (29 from Kodungallur and eight from Thuravoor) were screened in the study, with the majority of farms (*n* = 25) having monoculture of *Litopenaeus vannamei* (whiteleg shrimp). The remaining 12 farms reared *Penaeus monodon* (tiger prawn) cultures. All the farms had been in operation for a minimum of 1 year at the time when sampling was conducted. Sizes of the farms ranged from 2 to 17 acres with at least two ponds in each farm. Samples mainly included shrimp, water, and sediment. All ponds stocked with shrimps were chosen for sampling. Water samples were collected in sterile sample containers (500 ml) from three different spots at some distance from the edge of the pond. These spots were selected in such a manner that influent and effluent points of the ponds were included. Sediment samples from the selected spots were scooped out and put in sterile plastic covers. An appropriate number of adult shrimps (3–4 nos) were collected using cast-net and transferred to sterile polythene bags and covered with ice. All the samples were labeled properly with information on farmer’s name, pond number, and date of collection. Samples were then brought to laboratory on ice (within 4–5 h).

### Sample Processing and Bacterial Isolation

All samples were processed at the Microbiology, Fermentation and Biotechnology Division of ICAR-Central Institute of Fisheries Technology (ICAR-CIFT), Cochin, Kerala. Water samples collected at different spots of the same pond were pooled together and homogenized by inverting the bottles several times. Similarly, sediment samples were also pooled and mixed well. After removing the carapace, shrimps were aseptically cut into head, body and tail and a mixture of these parts were taken for bacterial isolation.

EE broth Mossel (BD Difco, United States), a modified form of brilliant green bile lactose broth was used for the selective enrichment of *Enterobacteriaceae*. Samples (10 ml of water; 10 g each of sediment and shrimp) were incubated in 90 ml of EE broth (pH 7.2) for 18–24 h at 37°C. To isolate ESBL-producing strains, a loopful of the enriched culture was streaked onto MacConkey agar plates (BD Difco, United States) supplemented with 1 μg/ml cefotaxime (Sigma-Aldrich, United States) and incubated for 18–24 h at 37°C. Lactose-fermenting colonies (pink in color) with morphological characteristics of *E. coli* or *K. pneumoniae* were picked and stored on tryptic soy agar (TSA) (BD Difco, United States) slants for identification and further characterization.

### Bacterial Identification and Antibiotic Susceptibility Testing (AST)

Bacterial species were identified using BD Phoenix^TM^ M50 automated system (BD Diagnostics, United States). The ID-AST combo panel, NMIC/ID55 designed for identification and susceptibility testing (MIC determination) of Gram-negative bacteria was used in this study. The panel consists of an ID side containing wells with dried substrates for bacterial identification and an AST side containing wells having varying concentrations of antimicrobial agents. With regard to ESBL detection, NMIC/ID55 panel has a “BD Phoenix ESBL screening test” which is based on the growth response of bacteria to selected second or third generation cephalosporins in the presence or absence of the beta-lactamase inhibitor, clavulanic acid. Procedures were performed according to manufacturer’s instructions. Briefly, bacterial colonies from pure cultures were transferred to the ID broth (BD Difco diagnostic systems, United States) and the inoculum density was adjusted to 0.5 McFarland using BD PhoenixSpec nephelometer. Twenty five microliters of the adjusted ID broth suspension was transferred to the AST broth with the AST indicator which is a resazurin-based dye. The suspensions (ID broth inoculum and AST broth inoculum) were then poured to the corresponding fill ports in the panel. Panels were sealed and loaded into the instrument for incubation at 35°C for around 16 h. Quality control was also performed using the reference strain *E. coli* ATCC 25922. BD Phoenix system is connected to EpiCenter, the data management software to analyze test results and generate reports. Further, BD Phoenix uses a rule-based expert system namely BDXpert which can interpret AST results and provide recommendations based on CLSI guidelines. Thus, on identifying specific resistance markers such as ESBL, BDXpert can alter, if necessary, the initial raw categorization (S,I, or R) for selected antibiotics and provide a “final SIR.” However, the MIC values are never altered.

### PCR Screening for Resistance Genes

Isolates of *E. coli* and *K. pneumoniae* alerted as “ESBL-producers” by BD Phoenix were screened for the presence of genes encoding various beta-lactamases. For this, DNA extraction was performed using DNeasy Blood and Tissue kits (Qiagen, Germany) as per manufacturer’s instructions. Two Multiplex PCRs (I and II) were employed for detecting CTX-M, TEM, SHV, and OXA-1-like genes using primers and amplification conditions as described by Dallenne et al. (2010). Multiplex I targeted CTX-M group 1, group 2, and group 9 enzymes, whereas multiplex II aimed at the simultaneous detection of *bla*_*TEM*_, *bla*_*SHV*_, and *bla*_*OXA*_ genes. Additionally, for positive isolates of CTX-M group 1, further PCR to detect the most common group 1 enzyme, *bla*_*CTX–M–15*_ was performed according to Gundran et al. (2019). Isolates were also tested for the presence of genes encoding AmpC enzymes by multiplex PCR that can detect the six major families of plasmid-mediated AmpC beta-lactamases (ACC, CIT, MOX, FOX, DHA, and EBC) (Dallenne et al., 2010). Presence of one of the most common pAmpC genes, *bla*_*CMY–2*_ (CIT-type) was also investigated using previously described primers (Kozak et al., 2009).

Further, based on phenotypic resistance patterns, PCRs were performed to identify various genes associated with resistance to different non-beta-lactam drugs. This included detection of genes conferring resistance to tetracycline (*tetA* and *tetB*), chloramphenicol (*cmlA* and *catA*), fluoroquinolones [*qnrA*, *qnrB*, *qnrS*, *qepA*, *oqxA*, *oqxB*, and *aac(6*′*)-Ib-cr*], aminoglycosides (*aadA1*, *strA*, and *strB*), and sulfonamides (*sul1* and *sul2*). All PCR reactions were carried out in a final volume of 25 μl containing 1X JumpStart RedTaq ReadyMix (Sigma-Aldrich, United States), primers in varying concentrations and 2 μl of DNA extract. PCR products were resolved on a 2% agarose gel containing ethidium bromide at a final concentration of 1 μg/ml. Details of the primers and PCR conditions used in this study for the detection of various resistance genes are given in the Supplementary Table 1.

### PCR-Based Replicon Typing (PBRT)

Plasmid incompatibility (InC) types were determined using the protocol described by Carattoli et al. (2005) which employs 18 primer pairs in five multiplex and three uniplex PCRs to distinguish the major InC groups (prevalent in *Enterobacteriaceae*) namely HI1, HI2, I1-Ic, X, L/M, N, FIA, FIB, W, Y, P, FIC, A/C, T, FIIAs, F, K, and B/O. All multiplex PCRs were run with the following conditions: initial denaturation at 94°C for 5 min, 30 cycles of denaturation at 94°C for 1 min, annealing at 60°C for 30 s and elongation at 72°C for 1 min and a final extension at 72°C for 5 min. Uniplex PCRs were also performed with the same conditions as above except that the annealing temperature was set at 52°C.

### Phylogenetic Grouping of ESBL-*E. coli* Isolates

Distribution of phylo-groups (A,B1,B2,C,D,E,F, and cryptic clade) among the ESBL-producing *E. coli* isolates was analyzed using the PCR method described by Clermont et al. (2013). This included an initial quadruplex PCR performed on the isolates to test the presence/absence of four genes: *arpA* (400 bp), *chuA* (288 bp), *yjaA* (211 bp), and *TspE4.C2* (152 bp). Based on the amplification pattern, an isolate either can be assigned to a specific phylogroup (B1, B2, and F) or needs additional PCRs to confirm the phylogroup (A,C,D,E, and clade I). All PCRs were performed with the following cycling conditions: initial denaturation at 94°C for 4 min, 30 cycles of denaturation at 94°C for 5 s, annealing at 59°C (quadruplex and group C) or 57°C (group E) for 20 s and extension at 72°C for 1 min, and a final extension at 72°C for 5 min.

### Detection of Virulence Genes in ESBL-*K. pneumoniae*

A multiplex PCR targeting the serotype-specific genes, *magA* (K1 serotype) and *wzi* (K2 serotype), and the virulence genes *rmpA*, *entB*, *ybtS*, *kfu*, *iutA*, *mrkD*, and *allS* was performed according to Compain et al. (2014). Cycling conditions included an initial denaturation at 95°C for 15 min, 30 cycles of denaturation at 94°C for 30 s, annealing at 60°C for 90 s and extension at 72°C for 60 s, and a final extension at 72°C for 10 min.

### Pulsed-Field Gel Electrophoresis (PFGE)

Pulsed-field gel electrophoresis was performed according to the standard operating procedure by PulseNet International^1^ to analyze the clonal relatedness between the isolates. Briefly, plugs containing whole genomic DNA of the *E. coli* or *K. pneumoniae* isolates were digested with *XbaI* (50 U/sample, New England Biolabs) at 37°C for 1.5–2 h. DNA fragments were separated on a 1% Megabase agarose gel (Bio-Rad, United States) in 0.5X TBE buffer at 14°C in a CHEF-Mapper XA device (Bio-Rad, United States). Electrophoresis conditions included a constant voltage of 6 V/cm, run time of 19 h, pulse time ranging from 6.76 to 35.38 s and an included angle of 120°. *Salmonella* serotype Braenderup H9812 was used as the standard molecular size marker. The gels were stained with ethidium bromide and observed under UV illumination. Gel images were exported to BioNumerics software package 7.6.3 (Applied Maths, Belgium) and cluster analysis was performed using the Unweighted Pair Group Method with Arithmetic mean (UPGMA) and Dice coefficient. Isolates which exhibited PFGE profiles with ≥85% similarity were considered genetically related.

## Results

### Sampling and Design Ethnographic Assessment

The present study analyzed shrimp (*n* = 77), water (*n* = 92), and sediment (*n* = 92) collected from a total of 37 farms. All the farmers consented to be interviewed and were willing to share the details of the culture and routine farming activities. Most of the farm owners were middle class with many of them having experience of working abroad and having access to other sources of income. Except for four farms which followed traditional farming practices, all other farms (*n* = 33) adhered to scientific farming. This included regular monitoring and management of water and soil quality, selection of good quality hatchery seeds for stocking, usage of commercially available high nutritive pelleted feeds, usage of aerators and harvesting only at the end of a crop season. On average, each farmer had 5.8 ± 4.5 (mean ± SD) acres of farm. Stocking density varied in the range between 30 and 60 nos/m^2^. Nearly 45% of the farmers in this study had experienced disease outbreaks in their farms. Infections caused by white spot syndrome virus (WSSV), a microsporidian parasite namely *Enterocytozoon Hepatopenaei* (EHP), and *Vibrio* bacteria were the most common. All the participating farmers reported that they hadn’t used any antibiotics in their farms. Probiotic usage was found to be a common practice among the farmers, with 62% of the farmers using at least one commercially available probiotic preparation. It is also noteworthy that natural remedies such as garlic paste and jaggery were cited by many farmers as supporting the health of their stock.

### Detection of ESBL-Producers and Their Resistance Profile

**FIGURE 2:**
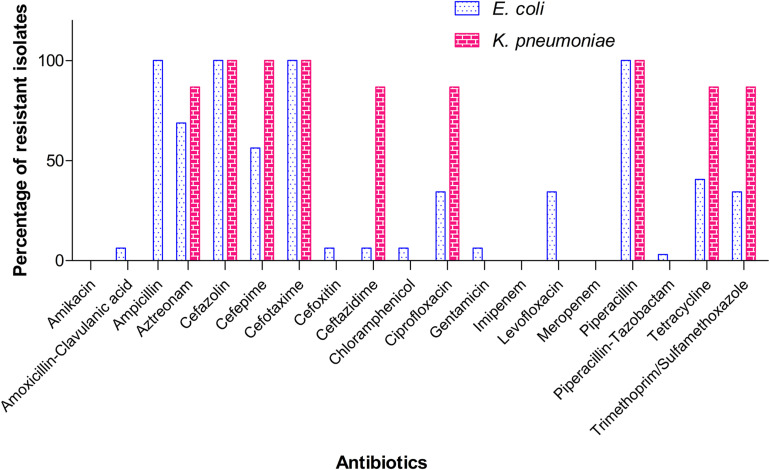
Resistance pattern of the ESBL-producing *E. coli* and *K. pneumoniae* isolates toward different antibiotics. *K. pneumoniae* is intrinsically resistant to ampicillin and therefore not shown here.

A total of 32 isolates of *E. coli* and 15 isolates of *K. pneumoniae* recovered from the samples were alerted as “ESBL-producers” by BD Phoenix^TM^ M50 automated system. Out of the 37 farms screened in the study, eight farms tested positive for ESBL- *E. coli* and five farms for *K. pneumoniae*. None of the farms harbored both the pathogens. Overall, five sediment samples and two each of shrimp and water samples yielded ESBL-*E. coli*, whereas for ESBL-*K. pneumoniae*, the following distribution was observed: sediment (0), shrimp (4), and water (1). Invariably all isolates of *E. coli* and *K. pneumoniae* from this study were resistant to ampicillin, piperacillin, cefazolin, and cefotaxime. MIC of cefotaxime was found to be ≥32 μg/ml for all isolates. However, MICs of other cephalosporins (ceftazidime and cefepime) and the monobactam drug, aztreonam varied among the isolates (Table 1). It is important to note that 18 (56.3%) *E. coli* isolates showed resistance (MIC ≥ 16 μg/ml) toward cefepime, a fourth generation cephalosporin and 14 (43.8%) isolates belonged to SDD category (susceptible-dose dependent, MIC 4–8 μg/ml). Notably, two isolates of *E. coli* were resistant to amoxicillin-clavulanate and intermediate to cefoxitin. In the case of *K. pneumoniae*, all isolates were resistant to cefepime (MIC ≥ 16 μg/ml). Percentage of resistance among *E. coli* isolates toward other antibiotics was as follows: tetracycline (40.6%), trimethoprim-sulfamethoxazole (34.4%), ciprofloxacin and levofloxacin (34.4%), chloramphenicol and gentamicin (6.3%). Of all isolates of *K. pneumoniae*, 13 (86.7%) showed resistance to tetracycline, ciprofloxacin, and trimethoprim/sulfamethoxazole (Figure 2). However, all ciprofloxacin-resistant isolates of *K. pneumoniae* remained susceptible to levofloxacin. Notably, multidrug resistance (resistance to at least one agent in ≥3 classes of antibiotics) was observed in all ESBL-positive isolates. Hundred per cent susceptibility was recorded for amikacin, imipenem, and meropenem drugs. MICs of all the tested antibiotics for *E. coli* and *K. pneumoniae* isolates are given in the Supplementary Tables 2, 3, respectively.

**TABLE 1:** Distribution of MICs of different cephalosporins and aztreonam among the ESBL-producing isolates.

Antibiotic	Cefazolin	Cefepime		Cefotaxime	Ceftazidime			Aztreonam		
					
MIC range (μ g/ml)	>16 (R)	4–8 (SDD)	≥16 (R)	≥32 (R)	4 (S)	8 (I)	≥16 (R)	4 (S)	8 (I)	≥16 (R)
**ESBL^+^ EC (n)**	32 (100%)	14 (43.8%)	18 (56.3%)	32 (100%)	13 (40.6%)	17 (53.1%)	2 (6.3%)	2 (6.3%)	11 (34.4%)	19 (59.4%)
**ESBL^+^ KP (n)**	15 (100%)	0	15 (100%)	15 (100%)	0	2 (13.3%)	13 (86.7%)	0	2 (13.3%)	13 (86.7%)

*ESBL^+^ EC, ESBL-producing *E. coli*; ESBL^+^ KP, ESBL-producing *K. pneumoniae*; R, resistant; I, intermediate; S, susceptible; SDD, susceptible dose dependent.*

### Detection of Resistance-Conferring Genes

**FIGURE 3:**
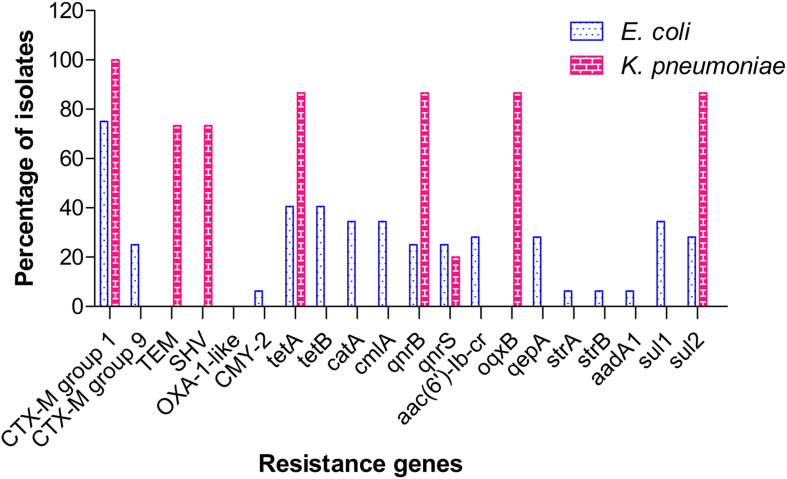
Distribution pattern of resistance genes among isolates of ESBL-producing *E. coli* and *K. pneumoniae.*

Among the ESBL genes screened, CTX-M group 1 was found to be the predominant type, with 23 *E. coli* (71.9%) and 15 *K. pneumoniae* (100%) isolates testing positive for the same. CTX-M group 9 was identified in 9 (28.1%) isolates of *E. coli*, but not in any of the *K. pneumoniae* isolates. Other beta-lactamase genes detected included blaTEM & blaSHV (11 *K. pneumoniae*, 73.3%) and blaCMY-2 (2 *E. coli*, 6.3%). No ESBL-positive isolate from our study carried OXA-1 type gene. Screening for tetracycline resistance genes (*tet*) identified the co-occurrence of *tetA* and *tetB* genes in 13 isolates (40.6%) of *E. coli*, whereas tetracycline resistance in *K. pneumoniae* isolates (13, 86.7%) was attributed to the presence of *tetA* alone. Other major resistance genes identified in *E. coli* included sulfonamide resistance genes, *sul1* (11, 34.4%) and *sul2* (9, 28.1%); chloramphenicol resistance genes, *catA*+*cmlA* (11, 34.4%); fluoroquinolone resistance genes, *qepA*+ *aac(6*′*)-Ib-cr* (9, 28.1%); and aminoglycoside resistance genes, *strA*+*strB*+*aadA1* (2, 6.25%). In contrast to *E. coli*, fluoroquinolone resistance in *K. pneumoniae* was mediated by *qnrB*+*oqxB* (13, 86.7%) and *qnrS* (3, 20%). Sulfonamide resistance in *K. pneumoniae* was attributed to *sul2* (13, 86.7%) alone. The distribution of resistance genes identified among the isolates is shown in Figure 3. A master chart detailing the phenotypic and genotypic characteristics of all isolates is provided as Supplementary Table 4.

### Replicon Typing

The predominant plasmid replicon types in *E. coli* were IncFIB and IncFIA, as observed in 21 (65.6%) and 15 (46.9%) isolates respectively. All isolates which carried IncFIA type had also harbored FIB type plasmids. However, six isolates were found to possess only FIB type plasmid. The employed primer sets could not determine plasmid types in 11 *E. coli* isolates. All isolates of ESBL-*K. pneumoniae* uniformly harbored IncHI1 type plasmids.

### Phylogenetic Grouping of ESBL-*E. coli* Isolates

Phylogenetic groups identified among the *E. coli* isolates included B1 (4, 12.5%), B2 (6, 18.8%), C (10, 31.3%), D (3, 9.4%), and E (9, 28.1%). With respect to the source of the isolates, the distribution of phylogroups was as follows: shrimp (B1, B2, and C), sediment (B1, B2, C, D, and E), and water (E).

### Detection of Virulence Genes in ESBL-*K. pneumoniae*

In the multiplex PCR employed to detect capsular serotypes K1 and K2, and seven major virulence factors, no K1- or K2-specific loci could be amplified in any of the *K. pneumoniae* isolates tested. Among the virulence-associated genes tested, *iutA*, *entB*, and *mrkD* were found in all the isolates.

### Molecular Typing by PFGE

**FIGURE 4:**
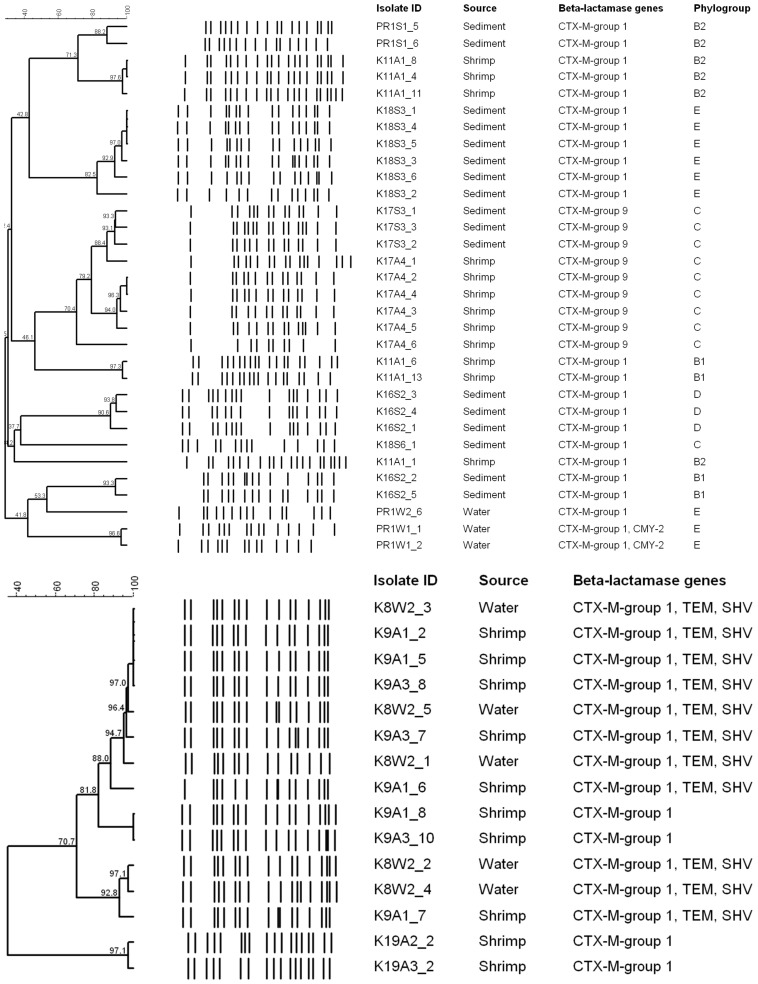
Dendrograms showing cluster analyses of *Xba1*-PFGE patterns of the ESBL-producing *E. coli* (top) and *K. pneumoniae* (below) isolates.

All isolates from this study were typeable by *XbaI*-PFGE. Applying a similarity cut-off value of 85%, isolates of *E. coli* (*n* = 32; 28 pulsotypes) and *K. pneumoniae* (*n* = 15, 11 pulsotypes) were grouped into 14 and four clusters, respectively (Figure 4). At this cut-off, five isolates of *E. coli*, namely K18S3_2, K17A4_6, K18S6_1, K11A1_1, and PR1W2_6 -all from different farms- did not cluster with other isolates. Overall, shrimp-, water-, and sediment-derived isolates of *E. coli* were scattered across 6, 7, and 2 clusters, respectively. Exclusively in one event, isolates of *E. coli* (K17A4_1, K17S3_1, K17S3_2, and K17S3_3) from two different farms were found clustered. In another instance, *E. coli* recovered from the water (PR1W1) and sediment (PR1S1) samples of the same farm were found genetically unrelated. In contrast to *E. coli*, isolates of *K. pneumoniae* from different sample types and/or farms were found clustered. The largest cluster had eight isolates recovered from both shrimp and water samples. All the four clusters of *K. pneumoniae* had shrimp-derived isolates; however, isolates recovered from water samples were grouped into two clusters.

## Discussion

There is a growing perception that aquaculture settings are contributing significantly to the development and dissemination of AMR. Although antibiotics are not used in aquaculture for growth promotion purposes, their prophylactic use is not uncommon in shrimp farms (Watts et al., 2017). A recent meta- analysis which studied data from nearly 40 countries, majority of which are LMICs, has shown that multi-antibiotic resistance index (MAR) of aquaculture-derived bacteria correlates strongly with that of human clinical isolates (Reverter et al., 2020). ESBL-producing *E. coli* is found widespread in clinical as well as other environments such as sewage, meat, food animals, etc. However, most of the studies on ESBL from India are confined to clinical settings, with little information available on the prevalence and epidemiology of ESBL-producing strains in aquaculture and other aquatic environments (Gandra et al., 2017). The low frequency of ESBL-positive isolates observed in our study could probably be attributed to the proper management of the ponds and general cleanliness maintained in the shrimp farms and surroundings. It is noteworthy that, in most cases, a given farm (which tested positive) had only one sample type -i.e., shrimp or sediment or water- yielding ESBL-positive isolate. Antibiotic usage appeared less-likely in the farms as all the farms we surveyed used commercial feeds certified as “antibiotic free” and most of the farmers were aware of the issues related to the imprudent use of antibiotics in aquaculture. However, the usage of any other supplements pre-mixed with antibiotics cannot be completely ruled out.

Though the majority of farms in our study tested negative for ESBL-producers, resistance traits observed in the isolates is a cause of concern. All isolates of *E. coli* and *K. pneumoniae* tested in our study were found to be resistant to at least four and five antibiotics, respectively. Moreover, nearly 41% of *E. coli* and 87% of *K. pneumoniae* isolates from this study were resistant to ≥8 out of the 19 antibiotics tested. In addition to beta-lactam and monobactam drugs to which ESBL-producing bacteria are generally resistant, 28.1% of *E. coli* isolates and 86.7% of *K. pneumoniae* isolates from our study showed simultaneous resistance to tetracycline, ciprofloxacin, and trimethoprim-sulfamethoxazole. Several previous studies reported increased frequency of bacteria resistant to tetracycline, fluoroquinolones and trimethoprim-sulfamethoxazole in aquaculture farms and surrounding regions (as reviewed by Cabello et al., 2013). Also, incidences of tetracycline-resistant *E. coli* and other bacterial spp. in shrimps imported from Asian countries have been reported (Ellis-Iversen et al., 2019; Khan et al., 2019). While acknowledging the fact that the major driver of AMR in aquaculture is the indiscriminate use of antibiotics in this sector, detection of resistant bacteria in aquaculture environment cannot always have direct link to the use of antimicrobials in the farm/setting in question. Aquatic systems are highly complex and dynamic in nature, and can receive effluents containing antibiotics/AMR bacteria from various other sources including hospitals, wastewater treatment plants, runoff from agricultural/livestock activities, etc. It is also possible that the source of the resistant bacteria is the hatchery where antibiotics are routinely used.

In recent years, *K. pneumoniae* has emerged as a major public health threat owing to high prevalence of MDR-strains producing ESBLs and/or carbapenemases. However, data pertaining to the occurrence, population structure and transmission routes of this pathogen in major animal, food and environmental reservoirs is quite limited (Chi et al., 2019; Wyres et al., 2020). In our study, multidrug resistance was observed at higher frequency in *K. pneumoniae* isolates when compared to *E. coli*. Moreover, higher MIC was observed for ceftazidime and cefepime in the majority of *K. pneumoniae* isolates. Similar to our observations, a previous study involving shrimp farms with intensive farming practices in Vietnam also reported multidrug-resistant isolates of *K. pneumoniae* (resistant mainly to sulfonamides, fluoroquinolones, and tetracycline) (Pham et al., 2018). On account of its broader ecological niche and high burden of plasmids, *K. pneumoniae* serves as a major transmitter of resistance from environment to various human or animal pathogens (Wyres and Holt, 2018).

Regarding the ESBL genotypes of our isolates, we observed the predominance of CTX-M-type ESBLs in both *E. coli* (100%) and *K. pneumoniae* (100%) followed by an equal representation of TEM and SHV enzymes in *K. pneumoniae* (73.3%). Thus, the majority (73.3%) of *K. pneumoniae* isolates from our study carried multiple beta-lactamases (*bla*_*CTX–M–15*_+ *bla*_*TEM*_ + *bla*_*SHV*_). As the primer set we employed in the present study detects both ESBL and non-ESBL variants of TEM and SHV enzymes, we cannot rule out the possibility of some of the TEM and SHV genes identified here being non-ESBL type. Co-occurrence of multiple ESBL determinants in *E. coli* or *K. pneumoniae* has been well documented in clinical settings (Hassan and Abdalhamid, 2014; Jena et al., 2017; Gautam et al., 2019). There is a paucity of data on the prevalence and molecular epidemiology of ESBL-producing bacteria from aquaculture environments; however, there are a couple of studies which reported the presence of ESBL genes in aquaculture settings or imported shrimps. In a study conducted on fishes from aquaculture farms in China, various ESBL genes such as *bla*_*CTX–M–14*,_ *bla*_*CTX–M–79*_, and *bla*_*SHV–27*_ were detected (Jiang et al., 2012). A previous study by Khan et al. (2019) reported CTX-M (56%) as the predominant ESBL gene, followed by TEM (16%) in *E. coli* isolates recovered from shrimps imported to United States from Asian countries. Similar incidence was also reported from Denmark where *E. coli* isolates from imported shrimps from Asia were found to harbor *bla*_*CTX–M–15*_ and *bla*_*CTX–M–55*_ genes (Ellis-Iversen et al., 2019). Many recent studies from Vietnam, where shrimp aquaculture is a major industry, reported ESBL-positive bacteria in retail shrimps and attributed this mainly to the inappropriate usage of antibiotics in shrimp farms (Le et al., 2015; Nguyen et al., 2016; Yen et al., 2020). Also, a study from Mumbai, India reported for the first time the detection of multiple ESBL genes in sea food isolates of *E. coli* (Singh et al., 2017).

Studies undertaken in various settings in India showed different patterns of distribution of ESBL genes. A study conducted in Vellore, South India reported high prevalence (91.8%) of CTX-M among ESBL-*E. coli* recovered from human (patients and healthy volunteers) and environmental samples (stagnant water bodies, sewage, public toilets, and meat markets) (George et al., 2015). In contrary, study by Bajaj et al. (2015) from north India reported the predominance of *bla*_*TEM*_ (100%) and low occurrence of *bla*_*CTX–M*_ (16%) among ESBL-*E. coli* recovered from aquatic environments. Nevertheless, similar to our findings, isolates from the above study also had *bla*_*CTX–M–15*_ as the CTX-M determinant. CTX-M-15, reported for the first time from India in 2011, has now become a widespread ESBL genotype and has been reported from humans, livestock and fishes (Palmeira and Ferreira, 2020). Importantly, two of our *E. coli* isolates harbored *bla*_*CMY–2*_, a CIT-type AmpC beta-lactamase. These isolates were resistant to cefotaxime, ceftazidime and amoxicillin-clavulanate, and intermediate towards cefoxitin. CMY-2 is one of the most common AmpC beta-lactamases encountered in *E. coli* isolated from humans and animals (Ewers et al., 2012). Co-expression of ESBL and AmpC has been reported previously in many environmental *E. coli* isolates (Bajaj et al., 2015; Ye et al., 2017).

In aquaculture, the prophylactic and therapeutic use of tetracycline has been well acknowledged and also shown to be a major factor contributing to the spread of tetracycline genes in the environment. Tetracycline resistance is mainly mediated by efflux pumps encoded by different *tet* genes, which often disseminate easily owing to their location on plasmids and transposons (Miranda et al., 2013). Further, these genes have been shown to persist in aquaculture settings even in the absence of selection pressure imposed by the continuous usage of tetracycline (Tamminen et al., 2011). All our tetracycline-resistant *E. coli* isolates carried both *tet(A)* and *tet(B)* genes and these isolates were from shrimp and sediment samples. Previous studies have also shown the predominance of *tet(A)*, *tet(B)*, and *tet(M)* genes among tetracycline-resistant isolates recovered from aquaculture farms and their environments (Akinbowale et al., 2007; Changkaew et al., 2014; Liyanage and Manage, 2019). Co-occurrence of *tetA* and *tetB* genes, as found in our study, was also reported in earlier studies carried out in aquaculture settings (Akinbowale et al., 2007; Nawaz et al., 2009). A study by Rhodes et al. (2000) showed that plasmids bearing tetracycline resistance determinants such as *tetA* disseminated between *Aeromonas* spp. and *E. coli* and between the aquaculture and human environments. This provides direct evidence that these settings interact more closely than previously thought. Our study also documents the prevalence of the sulfonamide resistance gene, *sul* and the chloramphenicol resistance genes, *catA* and *cmlA*. *Sul1* has been shown to be a potential indicator for assessing contamination by antibiotic resistance genes (ARGs) in aquaculture environments (Su et al., 2017). Recently Wang et al. (2019) reported predominance of *sul1* and *cmlA* among various antibiotic resistance genes (ARGs) in the rearing environments of intensive shrimp farms in South China.

The other major resistance trait among our isolates was the fluoroquinolone resistance, observed in 34.4% of *E. coli* and 73.3% *K. pneumoniae* isolates. Similar to our findings, a study from North India reported co-resistance to fluoroquinolones among ESBL-producing *E. coli* from aquatic sources (Bajaj et al., 2016). Further, a meta-analysis by Wiener et al. (2016) also reported significant association between ESBL phenotype and fluoroquinolone resistance in *Enterobacteriaceae* and cautions the empirical use of quinolones in treating ESBL infections. In our study, quinolone resistance in *E. coli* isolates was found mediated by both *qepA* and *aac(6*′*)-Ib-cr* which encode, respectively, a multidrug efflux pump and a mutant aminoglycoside-modifying enzyme capable of modifying quinolones as well. However, in *K. pneumoniae* isolates, *qnr genes (qnrB* and *qnrS)* which code for proteins that protect the target enzyme from quinolones action and the efflux pump-encoding *oqxB* gene accounted for resistance. A recent study from Kerala which investigated the prevalence of quinolone-resistant *E. coli* in water bodies contaminated with hospital effluents and in the surrounding aquaculture farms reported the predominance of *qnrB* followed by *qnrS*, *oqxAB*, *qnrA*, and *aac (6’)-Ib-cr* (Girijan et al., 2020). Plasmid-mediated quinolone resistance (PMQR) is a growing concern owing to the fact that fluoroquinolones are one of the safer and widely prescribed drugs and the mainstay for treatment of serious Gram-negative infections. PMQR genes such as *qnrA* and *qnrS* are shared between the aquatic *Shewanella*, *Aeromonas*, *Vibrio* and the human pathogens *E. coli* and *K. pneumoniae*, suggesting that many ARGs originated in aquatic environments before their dissemination to terrestrial hosts (Poirel et al., 2012).

Phylogenetic grouping showed that *E. coli* isolates recovered from shrimp samples belonged to B1, B2, and C. This is a worrisome finding from public health point of view as most of the virulent extra-intestinal strains of *E. coli* are primarily from group B2 and to a lesser extent from group D. In a study from Vietnam, B1 was found to be the most prevalent phylogroup in ESBL-*E. coli* isolated from retail shrimps (Le et al., 2015). It has been suggested that B2 *E. coli* isolates are more sensitive toward antibiotics, particularly to quinolones compared to non-B2 *E. coli* isolates (Skurnik et al., 2009). This was evident in our B2 isolates all of which were sensitive to quinolones, tetracycline, and trimethoprim-sulfamethoxazole. The majority of fluoroquinoline-resistant *E. coli* isolates from this study belonged to phylogroup C. With regard to the association of CTX-M group with phylogroups, we noted that CTX-M-15-producing isolates were spread over different phylogroups viz., B1, B2, C, D, and E, whereas all CTX-M-group 9 isolates belonged to phylogroup C.

Concerning the distribution of plasmids, we have found IncF and IncHI1 as the major replicon types among the isolates of *E. coli* and *K. pneumoniae*, respectively. A previous study had reported B/O as the predominant replicon type, followed by FIA in ESBL-*E. coli* isolated from imported shrimp (Khan et al., 2019). It has been shown that resistance genes, harbored on the narrow host range IncF-type plasmids in *Enterobacteriaceae* spread readily in *E. coli* (Mathers et al., 2015). Moreover, IncF plasmids carrying *bla*_*CTX–M–15*_ have been found in *Enterobacteriaceae* isolated from clinical, environmental, and livestock settings (Zurfluh et al., 2015). This perhaps indicates the role of IncF plasmids in disseminating CTX-M-15 across different settings. IncHI1, the plasmid type found in the *K. pneumoniae* isolates from our study, generally have broad host range and have been reported in various environmental Gram-negative species (Villa et al., 2012). However, many recent studies including one from India reported IncFII_*k*_ and other IncF plasmids as carriers of *bla*_*CTX–M–15*_ in *K. pneumoniae* (Shankar et al., 2018; Kakuta et al., 2020). In regard to virulence genes, our *K. pneumoniae* isolates harbored the siderophore genes *entB* (enterobactin) and *iutA* (aerobactin), and the type 3 fimbriae-encoding gene *mrkD*. Siderophore systems consist of extracellular iron-chelating molecules capable of scavenging Fe^3+^ from host proteins, and surface receptors for internalization. Enterobactin is a core siderophore present ubiquitously in *K. pneumoniae*, whereas, aerobactin is an acquired siderophore occasionally encountered in *Klebsiella* with an incidence rate of <10% (El Fertas-Aissani et al., 2013). Type 3 fimbriae, another frequent virulence determinant found in clinical as well as environmental isolates of *K. pneumoniae* aid in intestinal colonization and are also promoters of biofilm formation on biotic and abiotic surfaces (Struve and Krogfelt, 2004; Khater et al., 2015).

Pulsed-field gel electrophoresis analysis revealed high heterogeneity in ESBL-*E. coli* isolates recovered from different shrimp farms. In most cases, multiple isolates from the same sample showed identical banding patterns; however, there were also instances where the same sample yielded isolates with unrelated PFGE profiles, indicating multiple sources contaminating the farm. These isolates also differed significantly with respect to their antibiogram and phylogroups. In a previous study by Jiang et al. (2019), high clonality was observed for *E. coli* isolates recovered from shrimps sold at different open markets in China owing to the fact that these shrimps were reared in the same large-scale shrimp farm. Concerning the *K. pneumoniae* isolates, considerable genetic relatedness was observed, with 86% (*n* = 13) of the isolates belonging to a single cluster at a 70% similarity cut-off. Presence of one dominant cluster might be indicative of a well-adapted clone in this environment.

In summary, our results indicate the presence of multidrug-resistant isolates of ESBL-producing *E. coli* and *K. pneumoniae*, with resistance mainly toward cephalosporins, fluoroquinolones, tetracycline and trimethoprim-sulfamethoxazole drugs, in samples from various shrimp aquaculture farms in Kerala. To our best knowledge, this study provides the first data on the molecular features of ESBL-producing isolates prevailing in shrimp aquaculture settings of this region. In our study, *bla*_*CTX–M*_ was found to be the predominant ESBL genotype in both *E. coli* and *K. pneumoniae*. This, along with a high prevalence of *tet, sul*, and PMQR genes may be a public health concern and emphasizes the need for monitoring aquaculture settings for the possible emergence of antibiotic-resistant bacteria. Though our investigation was limited to a few numbers of farms and the results may only indicate a local trend, it has important implications for public health considering the long and complex nature of the shrimp supply chain starting from the farm worker to the consumer. Moreover, shrimp being an internationally traded commodity, can potentially aid in the transmission of resistant bacteria to different geographical regions.

## Data Availability Statement

The original contributions presented in the study are included in the article/Supplementary Material, further inquiries can be directed to the corresponding authors.

## Author Contributions

GKS, RE, TTB, and AP conceived the study and designed the experiments. VR and AV collected the samples and performed the experiments. GKS and VR analyzed and interpreted the results. VR wrote the manuscript. GKS and AP reviewed the manuscript. All authors contributed to the article and approved the submitted version.

## Conflict of Interest

The authors declare that the research was conducted in the absence of any commercial or financial relationships that could be construed as a potential conflict of interest.

## Publisher’s Note

All claims expressed in this article are solely those of the authors and do not necessarily represent those of their affiliated organizations, or those of the publisher, the editors and the reviewers. Any product that may be evaluated in this article, or claim that may be made by its manufacturer, is not guaranteed or endorsed by the publisher.

## Footnotes

1
https://www.cdc.gov/pulsenet/pdf/ecoli-shigella-salmonella-pfge-protocol-508c.pdf
