# Supplementary material for: Antibiotic Resistance Profiles and Molecular Characteristics of Extended-Spectrum Beta-Lactamase (ESBL)-Producing Escherichia coli and Klebsiella pneumoniae Isolated From Shrimp Aquaculture Farms in Kerala, India
Source: Front Microbiol. 2021 Aug 19;12:622891. doi: 10.3389/fmicb.2021.622891 (PMC8417373; doi:10.3389/fmicb.2021.622891)
Supplement: Supplementary file 1 [file Data_Sheet_1.docx]

**Table S1**. Details of the primers and PCR conditions used in this study for the detection of various antibiotic resistance genes

| Resistance phenotype | Resistance gene | Primer sequence (5'-3')  Forward Reverse | | PCR type | Amplicon size  (bp) | T_A_  (°C) | Reference |
| --- | --- | --- | --- | --- | --- | --- | --- |
| ESBL | CTX-M gp 1  CTX-M gp 2  CTX-M gp 9 | TTAGGAARTGTGCCGCTGYA  CGTTAACGGCACGATGAC  TCAAGCCTGCCGATCTGGT | CGATATCGTTGGTGGTRCCAT  CGATATCGTTGGTGGTRCCAT  TGATTCTCGCCGCTGAAG | Multiplex^a^ | 688  404  561 | 60 | Dallenne et al., 2010 |
| ESBL | TEM  SHV  OXA-1-like | CATTTCCGTGTCGCCCTTATTC  AGCCGCTTGAGCAAATTAAAC  GGCACCAGATTCAACTTTCAAG | CGTTCATCCATAGTTGCCTGAC  ATCCCGCAGATAAATCACCAC  GACCCCAAGTTTCCTGTAAGTG | Multiplex^a^ | 800  713  564 | 60 | Dallenne et al., 2010 |
| AmpC | ACC  FOX  MOX  DHA  CIT  EBC | CACCTCCAGCGACTTGTTAC  CTACAGTGCGGGTGGTTT  GCAACAACGACAATCCATCCT  TGATGGCACAGCAGGATATTC  CGAAGAGGCAATGACCAGAC  CGGTAAAGCCGATGTTGCG | GTTAGCCAGCATCACGATCC  CTATTTGCGGCCAGGTGA  GGGATAGGCGTAACTCTCCCAA  GCTTTGACTCTTTCGGTATTCG  ACGGACAGGGTTAGGATAGY  AGCCTAACCCCTGATACA | Multiplex^a^ | 346  162  895  997  538  683 | 60 | Dallenne et al., 2010 |
| AmpC | CMY-2 | GACAGCCTCTTTCTCCACA | TGGACACGAAGGCTACGTA | Uniplex^b^ | 1000 | 55 | Kozak et al., 2009 |
| Tetracycline | *tetA*  *tetB* | GGTTCACTCGAACGACGTCA  CCTCAGCTTCTCAACGCGTG | CTGTCCGACAAGTTGCATGA  GCACCTTGCTGATGACTCTT | Uniplex^c^ | 577  634 | 56  56 | Momtaz et al., 2012 |
| Chloramph-enicol | *cat1*  *cmlA* | AGTTGCTCAATGTACCTATAACC  CCGCCACGGTGTTGTTGTTATC | TTGTAATTCATTAAGCATTCTGCC  CACCTTGCCTGCCCATCATTAG | Uniplex^d^ | 547  698 | 55  55 | Momtaz et al., 2012 |
| Fluoroquinolone | *qnrA*  *qnrB*  *qnrS* | ATTTCTCACGCCAGGATTTG  GATCGTGAAAGCCAGAAAGG  GCAAGTTCATTGAACAGGGT | GATCGGCAAAGGTTAGGTCA  ATGAGCAACGATGCCTGGTA  TCTAAACCGTCGAGTTCGGCG | Multiplex^e^ | 516  476  428 | 56 | Kar et al., 2015 |
| Fluoroquinolone | *qepA*  *OqxA*  *oqxB* | CGTGTTGCTGGAGTTCTTC  GACAGCGTCGCACAGAATG  CGAAGAAAGACCTCCCTACCC | CTGCAGGTACTGCGTCATG  GGAGACGAGGTTGGTATGGA  CGCCGCCAATGAGATACA | Uniplex^f^ | 403  339  240 | 57  54  54 | Cattoir et al., 2008  Chen et al., 2012  Chen et al., 2012 |
| Fluoroquinolone | *aac(6')-Ib-cr* | TTGCGATGCTCTATGAGTGGCTA | CTCGAATGCCTGGCGTGTTT | Uniplex^g^ | 482 | 55 | Park et al., 2006 |
| Aminoglycoside | *StrA*  *StrB*  *aadA1* | CCTGGTGATAACGGCAATTC  ATCGTCAAGGGATTGAAACC  TATCCAGCTAAGCGCGAACT | CCAATCGCAGATAGAAGGC  GGATCGTAGAACATATTGGC  ATTTGCCGACTACCTTGGTC | Uniplex^h^ | 546  509  447 | 55  55  58 | Boerlin et al., 2005  Boerlin et al., 2005  Momtaz et al., 2012 |
| Sulfonamide | *sul1*  *sul2* | CGGCGTGGGCTACCTGAACG  GCGCTCAAGGCAGATGGCATT | GCCGATCGCGTGAAGTTCCG  GCGTTTGATACCGGCACCCGT | Multiplex^i^ | 433  293 | 67 | Gündoğdu et al., 2011 |

^a^Multiplex PCRs were performed with the following cycling conditions: Initial denaturation at 94 °C for 10 min; 30 cycles of denaturation at 94 °C for 30 S, annealing at 60 °C for 40 S and extention at 72 °C for 1 min; final extension at 72 °C for 7 min

^b^Cycling conditions: 94 °C for 15 min; 30 cycles of denaturation at 94 °C for 1 min, annealing at 55 °C for 1 min and extention at 72 °C for 1 min; final extension at 72 °C for 10 min

^c^Cycling conditions: 95 °C for 3 min; 35 cycles of denaturation at 94 °C for 1 min, annealing at 56 °C for 90 S and extention at 72 °C for 1 min; final extension at 72 °C for 8 min

^d^Cycling conditions: 95 °C for 3 min; 35 cycles of denaturation at 94 °C for 1 min, annealing at 55 °C for 90 S and extention at 72 °C for 1 min; final extension at 72 °C for 8 min

^e^Cycling conditions: 95 °C for 5 min; 35 cycles of denaturation at 95 °C for 30 S, annealing at 56 °C for 1 min and extention at 72 °C for 1 min; final extension at 72 °C for 10 min

^f^Cycling conditions: 94 °C for 10 min; 35 cycles of denaturation at 94 °C for 1 min, annealing temperature for 1 min and extention at 72 °C for 1 min; final extension at 72 °C for 10 min

^g^Cycling conditions: 94 °C for 10 min; 34 cycles of denaturation at 94 °C for 45 S, annealing at 55 °C for 45 S and extention at 72 °C for 45 S; final extension at 72 °C for 7 min

^h^Cycling conditions: 95 °C for 4 min; 35 cycles of denaturation at 94 °C for 1 min, annealing temperature for 1 min and extention at 72 °C for 1 min; final extension at 72 °C for 8 min

^i^Cycling conditions: 94 °C for 5 min; 36 cycles of denaturation at 94 °C for 15 S, 67 °C for 30 S and extension at 72 °C for 1 min; final extension at 72 °C for 7 min

**Table S2**. Antibiogram and MIC distribution determined for the isolates of ESBL-producing *E. coli* from the present study

|  | **Antibiotic susceptibility pattern & MIC (µg/ml)** | | | | | | | | | | | | | | | | | | |
| --- | --- | --- | --- | --- | --- | --- | --- | --- | --- | --- | --- | --- | --- | --- | --- | --- | --- | --- | --- |
| **Isolate**  **(Source)** | **AK** | **AMC** | **AMP** | **AZT** | **CFZ** | **CPM** | **CTX** | **CX** | **CAZ** | **CHL** | **CIP** | **GEN** | **IP** | **LE** | **MP** | **PIP** | **PTZ** | **TET** | **SXT** |
| K11A1-1  (Shrimp) | S | S | R | R | R | R | R | S | I | S | S | S | S | S | S | R | S | S | S |
|  | <=8 | 8/4 | >16 | >16 | >16 | 16 | >32 | <=4 | 8 | <=4 | <=0.5 | <=2 | <=1 | <=1 | <=1 | >64 | <=4/4 | <=2 | <=0.5/9.5 |
| K11A1-4  (Shrimp) | S | S | R | R | R | R | R | S | I | S | S | S | S | S | S | R | S | S | S |
|  | <=8 | 8/4 | >16 | >16 | >16 | 16 | >32 | <=4 | 8 | <=4 | <=0.5 | <=2 | <=1 | <=1 | <=1 | >64 | <=4/4 | <=2 | <=0.5/9.5 |
| K11A1-6  (Shrimp) | S | S | R | R | R | R | R | S | I | R | S | R | S | S | S | R | S | R | R |
|  | <=8 | 8/4 | >16 | >16 | >16 | >16 | >32 | <=4 | 8 | >16 | <=0.5 | >8 | <=1 | <=1 | <=1 | >64 | <=4/4 | >8 | >2/38 |
| K11A1-8  (Shrimp) | S | S | R | R | R | R | R | S | I | S | S | S | S | S | S | R | S | S | S |
|  | <=8 | 8/4 | >16 | >16 | >16 | 16 | >32 | <=4 | 8 | <=4 | <=0.5 | <=2 | <=1 | <=1 | <=1 | >64 | <=4/4 | <=2 | <=0.5/9.5 |
| K11A1-11  (Shrimp) | S | S | R | R | R | R | R | S | I | S | S | S | S | S | S | R | S | S | S |
|  | <=8 | 8/4 | >16 | >16 | >16 | 16 | >32 | <=4 | 8 | <=4 | <=0.5 | <=2 | <=1 | <=1 | <=1 | >64 | <=4/4 | <=2 | <=0.5/9.5 |
| K11A1-13  (Shrimp) | S | S | R | R | R | R | R | S | I | R | S | R | S | S | S | R | S | R | R |
|  | <=8 | 8/4 | >16 | >16 | >16 | >16 | >32 | <=4 | 8 | >16 | <=0.5 | >8 | <=1 | <=1 | <=1 | >64 | <=4/4 | >8 | >2/38 |
| K17A4-1  (Shrimp) | S | S | R | I | R | SDD | R | S | S | I | R | S | S | R | S | R | S | R | R |
|  | <=8 | <=4/2 | >16 | 8 | >16 | 4 | 32 | 8 | 4 | 16 | >2 | <=2 | <=1 | >4 | <=1 | >64 | <=4/4 | >8 | >2/38 |
| K17A4-2  (Shrimp) | S | S | R | I | R | SDD | R | S | S | I | R | S | S | R | S | R | S | R | R |
|  | <=8 | <=4/2 | >16 | 8 | >16 | 4 | >32 | 8 | 4 | 16 | >2 | <=2 | <=1 | >4 | <=1 | >64 | <=4/4 | >8 | >2/38 |
| K17A4-3  (Shrimp) | S | S | R | I | R | SDD | R | S | S | I | R | S | S | R | S | R | S | R | R |
|  | <=8 | <=4/2 | >16 | 8 | >16 | 4 | 32 | <=4 | 4 | 16 | >2 | <=2 | <=1 | >4 | <=1 | >64 | <=4/4 | >8 | >2/38 |
| K17A4-4  (Shrimp) | S | S | R | I | R | SDD | R | S | S | I | R | S | S | R | S | R | S | R | R |
|  | <=8 | <=4/2 | >16 | 8 | >16 | 4 | 32 | <=4 | 4 | 16 | >2 | <=2 | <=1 | >4 | <=1 | >64 | <=4/4 | >8 | >2/38 |
| K17A4-5  (Shrimp) | S | S | R | I | R | SDD | R | S | S | I | R | S | S | R | S | R | S | R | R |
|  | <=8 | <=4/2 | >16 | 8 | >16 | 4 | 32 | <=4 | 4 | 16 | >2 | <=2 | <=1 | >4 | <=1 | >64 | <=4/4 | >8 | >2/38 |
| K17A4-6  (Shrimp) | S | S | R | I | R | SDD | R | S | S | I | R | S | S | R | S | R | S | R | R |
|  | <=8 | <=4/2 | >16 | 8 | >16 | 4 | 32 | <=4 | 4 | 16 | >2 | <=2 | <=1 | >4 | <=1 | >64 | <=4/4 | >8 | >2/38 |
| K17S3-1  (Sediment) | S | S | R | S | R | SDD | R | S | S | I | R | S | S | R | S | R | S | R | R |
|  | <=8 | <=4/2 | >16 | 4 | >16 | 4 | 32 | 8 | 4 | 16 | >2 | <=2 | <=1 | >4 | <=1 | >64 | <=4/4 | >8 | >2/38 |
| K17S3-2  (Sediment) | S | S | R | S | R | SDD | R | S | S | I | R | S | S | R | S | R | S | R | R |
|  | <=8 | <=4/2 | >16 | 4 | >16 | 4 | 32 | 8 | 4 | 16 | >2 | <=2 | <=1 | >4 | <=1 | 64 | <=4/4 | >8 | >2/38 |
| K17S3-3  (Sediment) | S | S | R | I | R | SDD | R | S | S | I | R | S | S | R | S | R | S | R | R |
|  | <=8 | <=4/2 | >16 | 8 | >16 | 4 | 32 | 8 | 4 | 16 | >2 | <=2 | <=1 | >4 | <=1 | 64 | <=4/4 | >8 | >2/38 |
| K16S2-1  (Sediment) | S | S | R | I | R | SDD | R | S | S | S | S | S | S | S | S | R | S | S | S |
|  | <=8 | <=4/2 | >16 | 8 | >16 | 4 | >32 | <=4 | 4 | 8 | <=0.5 | <=2 | <=1 | <=1 | <=1 | >64 | <=4/4 | <=2 | <=0.5/9.5 |
| K16S2-2  (Sediment) | S | S | R | R | R | R | R | S | S | S | S | S | S | S | S | R | S | S | S |
|  | <=8 | <=4/2 | >16 | 16 | >16 | >16 | >32 | <=4 | 4 | <=4 | <=0.5 | <=2 | <=1 | <=1 | <=1 | >64 | <=4/4 | <=2 | <=0.5/9.5 |
| K16S2-3  (Sediment) | S | S | R | I | R | SDD | R | S | S | S | S | S | S | S | S | R | S | S | S |
|  | <=8 | <=4/2 | >16 | 8 | >16 | 8 | >32 | <=4 | 4 | 8 | <=0.5 | <=2 | <=1 | <=1 | <=1 | >64 | <=4/4 | <=2 | <=0.5/9.5 |
| K16S2-4  (Sediment) | S | S | R | I | R | SDD | R | S | S | S | S | S | S | S | S | R | S | S | S |
|  | <=8 | <=4/2 | >16 | 8 | >16 | 4 | >32 | <=4 | 4 | 8 | <=0.5 | <=2 | <=1 | <=1 | <=1 | >64 | <=4/4 | <=2 | <=0.5/9.5 |
| K16S2-5  (Sediment) | S | S | R | R | R | R | R | S | I | S | S | S | S | S | S | R | S | S | S |
|  | <=8 | <=4/2 | >16 | 16 | >16 | >16 | >32 | <=4 | 8 | <=4 | <=0.5 | <=2 | <=1 | <=1 | <=1 | >64 | <=4/4 | <=2 | <=0.5/9.5 |
| K18S3-1  (Sediment) | S | S | R | R | R | SDD | R | S | I | S | S | S | S | S | S | R | S | S | S |
|  | <=8 | <=4/2 | >16 | 16 | >16 | 8 | >32 | <=4 | 8 | 8 | <=0.5 | <=2 | <=1 | <=1 | <=1 | >64 | <=4/4 | <=2 | <=0.5/9.5 |
| K18S3-2  (Sediment) | S | S | R | R | R | R | R | S | I | S | S | S | S | S | S | R | S | S | S |
|  | <=8 | <=4/2 | >16 | 16 | >16 | 16 | 32 | <=4 | 8 | 8 | <=0.5 | <=2 | <=1 | <=1 | <=1 | >64 | <=4/4 | <=2 | <=0.5/9.5 |
| K18S3-3  (Sediment) | S | S | R | R | R | R | R | S | I | S | S | S | S | S | S | R | S | S | S |
|  | <=8 | <=4/2 | >16 | 16 | >16 | >16 | >32 | <=4 | 8 | 8 | <=0.5 | <=2 | <=1 | <=1 | <=1 | >64 | <=4/4 | <=2 | <=0.5/9.5 |
| K18S3-4  (Sediment) | S | S | R | R | R | R | R | S | I | S | S | S | S | S | S | R | S | S | S |
|  | <=8 | <=4/2 | >16 | 16 | >16 | 16 | >32 | <=4 | 8 | 8 | <=0.5 | <=2 | <=1 | <=1 | <=1 | >64 | <=4/4 | <=2 | <=0.5/9.5 |
| K18S3-5  (Sediment) | S | S | R | R | R | R | R | S | I | S | S | S | S | S | S | R | S | S | S |
|  | <=8 | <=4/2 | >16 | 16 | >16 | 16 | >32 | <=4 | 8 | 8 | <=0.5 | <=2 | <=1 | <=1 | <=1 | >64 | <=4/4 | <=2 | <=0.5/9.5 |
| K18S3-6  (Sediment) | S | S | R | R | R | SDD | R | S | I | S | S | S | S | S | S | R | S | S | S |
|  | <=8 | <=4/2 | >16 | 16 | >16 | 8 | 32 | <=4 | 8 | 8 | <=0.5 | <=2 | <=1 | <=1 | <=1 | >64 | <=4/4 | <=2 | <=0.5/9.5 |
| K18S6-1  (Sediment) | S | S | R | R | R | R | R | S | I | S | S | S | S | S | S | R | S | S | S |
|  | <=8 | <=4/2 | >16 | 8 | >16 | 16 | >32 | <=4 | 8 | 8 | <=0.5 | <=2 | <=1 | <=1 | <=1 | >64 | <=4/4 | <=2 | <=0.5/9.5 |
| PR1W1-1  (Water) | S | R | R | R | R | R | R | I | R | S | R | S | S | R | S | R | I | R | S |
|  | <=8 | >16/8 | >16 | >16 | >16 | >16 | >32 | 16 | 16 | <=4 | >2 | <=2 | <=1 | 4 | <=1 | >64 | 32/4 | >8 | <=0.5/9.5 |
| PR1W1-2  (Water) | S | R | R | R | R | R | R | I | R | S | R | S | S | R | S | R | R | R | S |
|  | <=8 | >16/8 | >16 | >16 | >16 | >16 | >32 | 16 | 16 | <=4 | >2 | <=2 | <=1 | 4 | <=1 | >64 | >64/4 | >8 | <=0.5/9.5 |
| PR1W2-6  (Water) | S | S | R | R | R | R | R | S | I | S | S | S | S | S | S | R | S | S | S |
|  | <=8 | <=4/2 | >16 | 16 | >16 | >16 | >32 | <=4 | 8 | <=4 | <=0.5 | <=2 | <=1 | <=1 | <=1 | >64 | <=4/4 | <=2 | <=0.5/9.5 |
| PR1S1-5  (Sediment) | S | S | R | R | R | R | R | S | I | S | S | S | S | S | S | R | S | S | S |
|  | <=8 | <=4/2 | >16 | 16 | >16 | >16 | >32 | <=4 | 8 | <=4 | <=0.5 | <=2 | <=1 | <=1 | <=1 | >64 | <=4/4 | <=2 | <=0.5/9.5 |
| PR1S1-6  (Sediment) | S | S | R | R | R | R | R | S | I | S | S | S | S | S | S | R | S | S | S |
|  | <=8 | <=4/2 | >16 | 16 | >16 | >16 | >32 | <=4 | 8 | <=4 | <=0.5 | <=2 | <=1 | <=1 | <=1 | >64 | <=4/4 | <=2 | <=0.5/9.5 |

**AK-**Amikacin; **AMC**-Amoxicillin-Clavulanic acid; **AMP**-Ampicillin; **AZT**-Aztreonam; **CFZ**-Cefazolin; **CPM**-Cefepime; **CTX**-Cefotaxime; **CX**-Cefoxitin; **CAZ**-Ceftazidime; **CHL**-Chlramphenicol; **CIP**-Ciprofloxacin; **GEN**-Gentamicin; **IP**-Imipenem; **LE**-Levofloxacin; **MP**-Meropenem; **PIP**-piperacillin; **PTZ**-Piperacillin-tazobactam; **TET**-Tetracycline; **SXT**-Trimethoprim-sulfamethoxazole

**R**-Resistant; **I**-Intermediate; **S**-Sensitive; **SDD**- Susceptible Dose Dependent

**Table S3.** Antibiogram and MIC distribution for the isolates of ESBL-producing *K. pneumoniae* from the present study

|  | **Antibiotic susceptibility pattern & MIC (µg/ml)** | | | | | | | | | | | | | | | | | | |
| --- | --- | --- | --- | --- | --- | --- | --- | --- | --- | --- | --- | --- | --- | --- | --- | --- | --- | --- | --- |
| **Isolate**  **(Source)** | **AK** | **AMC** | **AMP** | **AZT** | **CFZ** | **CPM** | **CTX** | **CX** | **CAZ** | **CHL** | **CIP** | **GEN** | **IP** | **LE** | **MP** | **PIP** | **PTZ** | **TET** | **SXT** |
| K9A3-8  (Shrimp) | S | S | R | R | R | R | R | S | R | S | R | S | S | S | S | R | S | R | R |
|  | <=8 | 8/4 | >16 | >16 | >16 | >16 | >32 | <=4 | >16 | <=4 | 1 | <=2 | <=1 | <=1 | <=1 | >64 | <=4/4 | >8 | >2/38 |
| K8W2-1  (Water) | S | S | R | R | R | R | R | S | R | S | R | S | S | S | S | R | S | R | R |
|  | <=8 | 8/4 | >16 | >16 | >16 | >16 | >32 | <=4 | >16 | <=4 | 1 | <=2 | <=1 | <=1 | <=1 | >64 | <=4/4 | >8 | >2/38 |
| K8W2-3  (Water) | S | S | R | R | R | R | R | S | R | S | R | S | S | S | S | R | S | R | R |
|  | <=8 | 8/4 | >16 | >16 | >16 | >16 | >32 | <=4 | >16 | <=4 | 1 | <=2 | <=1 | <=1 | <=1 | >64 | <=4/4 | >8 | >2/38 |
| K9A3-7  (Shrimp) | S | S | R | R | R | R | R | S | R | S | R | S | S | S | S | R | S | R | R |
|  | <=8 | 8/4 | >16 | >16 | >16 | >16 | >32 | <=4 | >16 | <=4 | 1 | <=2 | <=1 | <=1 | <=1 | >64 | <=4/4 | >8 | >2/38 |
| K9A1-6  (Shrimp) | S | I | R | R | R | R | R | S | R | S | R | S | S | S | S | R | S | R | R |
|  | <=8 | 16/8 | >16 | >16 | >16 | >16 | >32 | <=4 | >16 | <=4 | 1 | <=2 | <=1 | <=1 | <=1 | >64 | <=4/4 | >8 | >2/38 |
| K9A1-5  (Shrimp) | S | S | R | R | R | R | R | S | R | S | R | S | S | S | S | R | S | R | R |
|  | <=8 | 8/4 | >16 | >16 | >16 | >16 | >32 | <=4 | >16 | <=4 | 1 | <=2 | <=1 | <=1 | <=1 | >64 | <=4/4 | >8 | >2/38 |
| K9A1-2  (Shrimp) | S | I | R | R | R | R | R | S | R | S | R | S | S | S | S | R | S | R | R |
|  | <=8 | 16/8 | >16 | >16 | >16 | >16 | >32 | <=4 | >16 | <=4 | 1 | <=2 | <=1 | <=1 | <=1 | >64 | <=4/4 | >8 | >2/38 |
| K9A1-8  (Shrimp) | S | I | R | R | R | R | R | S | R | S | R | S | S | S | S | R | S | R | R |
|  | <=8 | 16/8 | >16 | >16 | >16 | >16 | >32 | <=4 | >16 | <=4 | 1 | <=2 | <=1 | <=1 | <=1 | >64 | <=4/4 | >8 | >2/38 |
| K8W2-4  (Water) | S | S | R | R | R | R | R | S | R | S | R | S | S | S | S | R | S | R | R |
|  | <=8 | 8/4 | >16 | >16 | >16 | >16 | >32 | <=4 | >16 | <=4 | 1 | <=2 | <=1 | <=1 | <=1 | >64 | <=4/4 | >8 | >2/38 |
| K8W2-2  (Water) | S | S | R | R | R | R | R | S | R | S | R | S | S | S | S | R | S | R | R |
|  | <=8 | 8/4 | >16 | >16 | >16 | >16 | >32 | <=4 | >16 | <=4 | 1 | <=2 | <=1 | <=1 | <=1 | >64 | <=4/4 | >8 | >2/38 |
| K8W2-5  (Water) | S | I | R | R | R | R | R | S | R | S | R | S | S | S | S | R | S | R | R |
|  | <=8 | 16/8 | >16 | >16 | >16 | >16 | >32 | <=4 | >16 | <=4 | 1 | <=2 | <=1 | <=1 | <=1 | >64 | <=4/4 | >8 | >2/38 |
| K9A3-10  (Shrimp) | S | S | R | R | R | R | R | S | R | S | R | S | S | S | S | R | S | R | R |
|  | <=8 | 8/4 | >16 | >16 | >16 | >16 | >32 | <=4 | >16 | <=4 | 1 | <=2 | <=1 | <=1 | <=1 | >64 | <=4/4 | >8 | >2/38 |
| K9A1-7  (Shrimp) | S | S | R | R | R | R | R | S | R | I | R | S | S | S | S | R | S | R | R |
|  | <=8 | 8/4 | >16 | >16 | >16 | >16 | >32 | <=4 | >16 | 16 | 1 | <=2 | <=1 | <=1 | <=1 | >64 | <=4/4 | >8 | >2/38 |
| K19A2-2  (Shrimp) | S | S | R | I | R | R | R | S | I | S | S | S | S | S | S | R | S | S | S |
|  | <=8 | <=4/2 | >16 | 8 | >16 | 16 | 32 | <=4 | 8 | <=4 | <=0.5 | <=2 | <=1 | <=1 | <=1 | >64 | <=4/4 | <=2 | <=0.5/9.5 |
| K19A3-2  (Shrimp) | S | S | R | I | R | R | R | S | I | S | S | S | S | S | S | R | S | S | S |
|  | <=8 | <=4/2 | >16 | 8 | >16 | 16 | 32 | <=4 | 8 | <=4 | <=0.5 | <=2 | <=1 | <=1 | <=1 | >64 | <=4/4 | <=2 | <=0.5/9.5 |

**AK-**Amikacin; **AMC**-Amoxicillin-Clavulanic acid; **AMP**-Ampicillin; **AZT**-Aztreonam; **CFZ**-Cefazolin; **CPM**-Cefepime; **CTX**-Cefotaxime; **CX**-Cefoxitin; **CAZ**-Ceftazidime; **CHL**-Chlramphenicol; **CIP**-Ciprofloxacin; **GEN**-Gentamicin; **IP**-Imipenem; **LE**-Levofloxacin; **MP**-Meropenem; **PIP**-piperacillin; **PTZ**-Piperacillin-tazobactam; **TET**-Tetracycline; **SXT**-Trimethoprim-sulfamethoxazole

**R**-Resistant; **I**-Intermediate; **S**-Sensitive

**Table S4**. Master chart showing the phenotypic and genotypic characteristics of the isolates of *E. coli* and *K. pneumoniae* from the present study

| **Isolate**  **ID** | **Organism** | | **Source** | | | **Beta-lactamases** | | | | | **Resistance pattern**  **Resistant; Intermediate; Susceptible; * SDD** | | | | | | | | | | | | | | | | | | | **Other resistance genes** | | | | | | | | | | | | | |
| --- | --- | --- | --- | --- | --- | --- | --- | --- | --- | --- | --- | --- | --- | --- | --- | --- | --- | --- | --- | --- | --- | --- | --- | --- | --- | --- | --- | --- | --- | --- | --- | --- | --- | --- | --- | --- | --- | --- | --- | --- | --- | --- | --- |
|  | **EC** | **KP** | **Shrimp** | **Water** | **Sediment** | **CTX-M-gp-1** | **CTX-M-gp-9** | **TEM** | **SHV** | **CMY-2** | **AK** | **AMC** | **AMP** | **AZT** | **CFZ** | **CPM** | **CTX** | **CX** | **CAZ** | **CHL** | **CIP** | **GEN** | **IP** | **LE** | **MP** | **PIP** | **PTZ** | **TET** | **SXT** | ***tetA*** | ***tetB*** | ***sul1*** | ***Sul2*** | ***qnrB*** | ***qnrS*** | ***qepA*** | ***oqxB*** | ***aac(6')-Ib-cr*** | ***cmlA*** | ***catA*** | ***strA*** | ***strB*** | ***aadA1*** |
| K11A1-1 |  |  |  |  |  |  |  |  |  |  |  |  |  |  |  |  |  |  |  |  |  |  |  |  |  |  |  |  |  |  |  |  |  |  |  |  |  |  |  |  |  |  |  |
| K11A1-4 |  |  |  |  |  |  |  |  |  |  |  |  |  |  |  |  |  |  |  |  |  |  |  |  |  |  |  |  |  |  |  |  |  |  |  |  |  |  |  |  |  |  |  |
| K11A1-6 |  |  |  |  |  |  |  |  |  |  |  |  |  |  |  |  |  |  |  |  |  |  |  |  |  |  |  |  |  |  |  |  |  |  |  |  |  |  |  |  |  |  |  |
| K11A1-8 |  |  |  |  |  |  |  |  |  |  |  |  |  |  |  |  |  |  |  |  |  |  |  |  |  |  |  |  |  |  |  |  |  |  |  |  |  |  |  |  |  |  |  |
| K11A1-11 |  |  |  |  |  |  |  |  |  |  |  |  |  |  |  |  |  |  |  |  |  |  |  |  |  |  |  |  |  |  |  |  |  |  |  |  |  |  |  |  |  |  |  |
| K11A1-13 |  |  |  |  |  |  |  |  |  |  |  |  |  |  |  |  |  |  |  |  |  |  |  |  |  |  |  |  |  |  |  |  |  |  |  |  |  |  |  |  |  |  |  |
| K16S2-1 |  |  |  |  |  |  |  |  |  |  |  |  |  |  |  | * |  |  |  |  |  |  |  |  |  |  |  |  |  |  |  |  |  |  |  |  |  |  |  |  |  |  |  |
| K16S2-2 |  |  |  |  |  |  |  |  |  |  |  |  |  |  |  |  |  |  |  |  |  |  |  |  |  |  |  |  |  |  |  |  |  |  |  |  |  |  |  |  |  |  |  |
| K16S2-3 |  |  |  |  |  |  |  |  |  |  |  |  |  |  |  | * |  |  |  |  |  |  |  |  |  |  |  |  |  |  |  |  |  |  |  |  |  |  |  |  |  |  |  |
| K16S2-4 |  |  |  |  |  |  |  |  |  |  |  |  |  |  |  | * |  |  |  |  |  |  |  |  |  |  |  |  |  |  |  |  |  |  |  |  |  |  |  |  |  |  |  |
| K16S2-5 |  |  |  |  |  |  |  |  |  |  |  |  |  |  |  |  |  |  |  |  |  |  |  |  |  |  |  |  |  |  |  |  |  |  |  |  |  |  |  |  |  |  |  |
| K17A4-1 |  |  |  |  |  |  |  |  |  |  |  |  |  |  |  | * |  |  |  |  |  |  |  |  |  |  |  |  |  |  |  |  |  |  |  |  |  |  |  |  |  |  |  |
| K17A4-2 |  |  |  |  |  |  |  |  |  |  |  |  |  |  |  | * |  |  |  |  |  |  |  |  |  |  |  |  |  |  |  |  |  |  |  |  |  |  |  |  |  |  |  |
| K17A4-3 |  |  |  |  |  |  |  |  |  |  |  |  |  |  |  | * |  |  |  |  |  |  |  |  |  |  |  |  |  |  |  |  |  |  |  |  |  |  |  |  |  |  |  |
| K17A4-4 |  |  |  |  |  |  |  |  |  |  |  |  |  |  |  | * |  |  |  |  |  |  |  |  |  |  |  |  |  |  |  |  |  |  |  |  |  |  |  |  |  |  |  |
| K17A4-5 |  |  |  |  |  |  |  |  |  |  |  |  |  |  |  | * |  |  |  |  |  |  |  |  |  |  |  |  |  |  |  |  |  |  |  |  |  |  |  |  |  |  |  |
| K17A4-6 |  |  |  |  |  |  |  |  |  |  |  |  |  |  |  | * |  |  |  |  |  |  |  |  |  |  |  |  |  |  |  |  |  |  |  |  |  |  |  |  |  |  |  |
| K17S3-1 |  |  |  |  |  |  |  |  |  |  |  |  |  |  |  | * |  |  |  |  |  |  |  |  |  |  |  |  |  |  |  |  |  |  |  |  |  |  |  |  |  |  |  |
| K17S3-2 |  |  |  |  |  |  |  |  |  |  |  |  |  |  |  | * |  |  |  |  |  |  |  |  |  |  |  |  |  |  |  |  |  |  |  |  |  |  |  |  |  |  |  |
| K17S3-3 |  |  |  |  |  |  |  |  |  |  |  |  |  |  |  | * |  |  |  |  |  |  |  |  |  |  |  |  |  |  |  |  |  |  |  |  |  |  |  |  |  |  |  |
| K18S3-1 |  |  |  |  |  |  |  |  |  |  |  |  |  |  |  | * |  |  |  |  |  |  |  |  |  |  |  |  |  |  |  |  |  |  |  |  |  |  |  |  |  |  |  |
| K18S3-2 |  |  |  |  |  |  |  |  |  |  |  |  |  |  |  |  |  |  |  |  |  |  |  |  |  |  |  |  |  |  |  |  |  |  |  |  |  |  |  |  |  |  |  |
| K18S3-3 |  |  |  |  |  |  |  |  |  |  |  |  |  |  |  |  |  |  |  |  |  |  |  |  |  |  |  |  |  |  |  |  |  |  |  |  |  |  |  |  |  |  |  |
| K18S3-4 |  |  |  |  |  |  |  |  |  |  |  |  |  |  |  |  |  |  |  |  |  |  |  |  |  |  |  |  |  |  |  |  |  |  |  |  |  |  |  |  |  |  |  |
| K18S3-5 |  |  |  |  |  |  |  |  |  |  |  |  |  |  |  |  |  |  |  |  |  |  |  |  |  |  |  |  |  |  |  |  |  |  |  |  |  |  |  |  |  |  |  |
| K18S3-6 |  |  |  |  |  |  |  |  |  |  |  |  |  |  |  | * |  |  |  |  |  |  |  |  |  |  |  |  |  |  |  |  |  |  |  |  |  |  |  |  |  |  |  |
| K18S6-1 |  |  |  |  |  |  |  |  |  |  |  |  |  |  |  |  |  |  |  |  |  |  |  |  |  |  |  |  |  |  |  |  |  |  |  |  |  |  |  |  |  |  |  |
| PR1W1-1 |  |  |  |  |  |  |  |  |  |  |  |  |  |  |  |  |  |  |  |  |  |  |  |  |  |  |  |  |  |  |  |  |  |  |  |  |  |  |  |  |  |  |  |
| PR1W1-2 |  |  |  |  |  |  |  |  |  |  |  |  |  |  |  |  |  |  |  |  |  |  |  |  |  |  |  |  |  |  |  |  |  |  |  |  |  |  |  |  |  |  |  |
| PR1W2-6 |  |  |  |  |  |  |  |  |  |  |  |  |  |  |  |  |  |  |  |  |  |  |  |  |  |  |  |  |  |  |  |  |  |  |  |  |  |  |  |  |  |  |  |
| PR1S1-5 |  |  |  |  |  |  |  |  |  |  |  |  |  |  |  |  |  |  |  |  |  |  |  |  |  |  |  |  |  |  |  |  |  |  |  |  |  |  |  |  |  |  |  |
| PR1S1-6 |  |  |  |  |  |  |  |  |  |  |  |  |  |  |  |  |  |  |  |  |  |  |  |  |  |  |  |  |  |  |  |  |  |  |  |  |  |  |  |  |  |  |  |
| K8W2-1 |  |  |  |  |  |  |  |  |  |  |  |  |  |  |  |  |  |  |  |  |  |  |  |  |  |  |  |  |  |  |  |  |  |  |  |  |  |  |  |  |  |  |  |
| K8W2-2 |  |  |  |  |  |  |  |  |  |  |  |  |  |  |  |  |  |  |  |  |  |  |  |  |  |  |  |  |  |  |  |  |  |  |  |  |  |  |  |  |  |  |  |
| K8W2-3 |  |  |  |  |  |  |  |  |  |  |  |  |  |  |  |  |  |  |  |  |  |  |  |  |  |  |  |  |  |  |  |  |  |  |  |  |  |  |  |  |  |  |  |
| K8W2-4 |  |  |  |  |  |  |  |  |  |  |  |  |  |  |  |  |  |  |  |  |  |  |  |  |  |  |  |  |  |  |  |  |  |  |  |  |  |  |  |  |  |  |  |
| K8W2-5 |  |  |  |  |  |  |  |  |  |  |  |  |  |  |  |  |  |  |  |  |  |  |  |  |  |  |  |  |  |  |  |  |  |  |  |  |  |  |  |  |  |  |  |
| K9A1-2 |  |  |  |  |  |  |  |  |  |  |  |  |  |  |  |  |  |  |  |  |  |  |  |  |  |  |  |  |  |  |  |  |  |  |  |  |  |  |  |  |  |  |  |
| K9A1-5 |  |  |  |  |  |  |  |  |  |  |  |  |  |  |  |  |  |  |  |  |  |  |  |  |  |  |  |  |  |  |  |  |  |  |  |  |  |  |  |  |  |  |  |
| K9A1-6 |  |  |  |  |  |  |  |  |  |  |  |  |  |  |  |  |  |  |  |  |  |  |  |  |  |  |  |  |  |  |  |  |  |  |  |  |  |  |  |  |  |  |  |
| K9A1-7 |  |  |  |  |  |  |  |  |  |  |  |  |  |  |  |  |  |  |  |  |  |  |  |  |  |  |  |  |  |  |  |  |  |  |  |  |  |  |  |  |  |  |  |
| K9A1-8 |  |  |  |  |  |  |  |  |  |  |  |  |  |  |  |  |  |  |  |  |  |  |  |  |  |  |  |  |  |  |  |  |  |  |  |  |  |  |  |  |  |  |  |
| K9A3-7 |  |  |  |  |  |  |  |  |  |  |  |  |  |  |  |  |  |  |  |  |  |  |  |  |  |  |  |  |  |  |  |  |  |  |  |  |  |  |  |  |  |  |  |
| K9A3-8 |  |  |  |  |  |  |  |  |  |  |  |  |  |  |  |  |  |  |  |  |  |  |  |  |  |  |  |  |  |  |  |  |  |  |  |  |  |  |  |  |  |  |  |
| K9A3-10 |  |  |  |  |  |  |  |  |  |  |  |  |  |  |  |  |  |  |  |  |  |  |  |  |  |  |  |  |  |  |  |  |  |  |  |  |  |  |  |  |  |  |  |
| K19A2-2 |  |  |  |  |  |  |  |  |  |  |  |  |  |  |  |  |  |  |  |  |  |  |  |  |  |  |  |  |  |  |  |  |  |  |  |  |  |  |  |  |  |  |  |
| K19A3-2 |  |  |  |  |  |  |  |  |  |  |  |  |  |  |  |  |  |  |  |  |  |  |  |  |  |  |  |  |  |  |  |  |  |  |  |  |  |  |  |  |  |  |  |

**AK-**Amikacin; **AMC**-Amoxicillin-Clavulanic acid; **AMP**-Ampicillin; **AZT**-Aztreonam; **CFZ**-Cefazolin; **CPM**-Cefepime; **CTX**-Cefotaxime; **CX**-Cefoxitin; **CAZ**-Ceftazidime; **CHL**-Chlramphenicol; **CIP**-Ciprofloxacin; **GEN**-Gentamicin; **IP**-Imipenem; **LE**-Levofloxacin; **MP**-Meropenem; **PIP**-piperacillin; **PTZ**-Piperacillin-tazobactam; **TET**-Tetracycline; **SXT**-Trimethoprim-sulfamethoxazole

**References**

Boerlin, P., Travis, R., Gyles, C.L., Reid-Smith, R., Lim, N.J.H., Nicholson, V., McEwen, S.A., Friendship, R. and Archambault, M., 2005. Antimicrobial resistance and virulence genes of *Escherichia coli* isolates from swine in Ontario. *Applied and environmental microbiology*, *71*(11), pp.6753-6761.

Cattoir, V., Poirel, L. and Nordmann, P., 2008. Plasmid-mediated quinolone resistance pump *QepA2* in an *Escherichia coli* isolate from France. *Antimicrobial agents and chemotherapy*, *52*(10), pp.3801-3804.

Chen, X., Zhang, W., Pan, W., Yin, J., Pan, Z., Gao, S. and Jiao, X., 2012. Prevalence of *qnr, aac (6′)-Ib-cr, qepA*, and *oqxAB* in *Escherichia coli* isolates from humans, animals, and the environment. *Antimicrobial agents and chemotherapy*, *56*(6), pp.3423-3427.

Dallenne, C., Da Costa, A., Decré, D., Favier, C. and Arlet, G., 2010. Development of a set of multiplex PCR assays for the detection of genes encoding important β-lactamases in Enterobacteriaceae. *Journal of Antimicrobial Chemotherapy*, *65*(3), pp.490-495.

Gündoğdu, A., Long, Y.B., Vollmerhausen, T.L. and Katouli, M., 2011. Antimicrobial resistance and distribution of sul genes and integron-associated intI genes among uropathogenic *Escherichia coli* in Queensland, Australia. *Journal of medical microbiology*, *60*(11), pp.1633-1642.

Kar, D., Bandyopadhyay, S., Bhattacharyya, D., Samanta, I., Mahanti, A., Nanda, P.K., Mondal, B., Dandapat, P., Das, A.K., Dutta, T.K. and Bandyopadhyay, S., 2015. Molecular and phylogenetic characterization of multidrug resistant extended spectrum beta-lactamase producing *Escherichia coli* isolated from poultry and cattle in Odisha, India. *Infection, Genetics and Evolution*, *29*, pp.82-90.

Kozak, G.K., Boerlin, P., Janecko, N., Reid-Smith, R.J. and Jardine, C., 2009. Antimicrobial resistance in *Escherichia coli* isolates from swine and wild small mammals in the proximity of swine farms and in natural environments in Ontario, Canada. *Applied and Environmental Microbiology*, *75*(3), pp.559-566.

Momtaz, H., Farzan, R., Rahimi, E., Safarpoor Dehkordi, F. and Souod, N., 2012. Molecular characterization of Shiga toxin-producing *Escherichia coli* isolated from ruminant and donkey raw milk samples and traditional dairy products in Iran. *The Scientific World Journal*, Article ID 231342.

Park, C.H., Robicsek, A., Jacoby, G.A., Sahm, D. and Hooper, D.C., 2006. Prevalence in the United States of *aac (6′)-Ib-cr* encoding a ciprofloxacin-modifying enzyme. *Antimicrobial agents and chemotherapy*, *50*(11), pp.3953-3955.
